# Association between vascular ultrasound features and DNA sequencing in breast cancer: a preliminary study

**DOI:** 10.1007/s12672-023-00657-8

**Published:** 2023-04-30

**Authors:** Mi-Ryung Han, Ah Young Park, Bo Kyoung Seo, Min Sun Bae, Jung Sun Kim, Gil Soo Son, Hye Yoon Lee, Young Woo Chang, Kyu Ran Cho, Sung Eun Song, Ok Hee Woo, Hye-Yeon Ju, Hyunseung Oh

**Affiliations:** 1 Division of Life Sciences, College of Life Sciences and Bioengineering, Incheon National University, Incheon, Republic of Korea; 2 Department of Radiology, CHA Bundang Medical Center, CHA University, Seongnam, Republic of Korea; 3 Department of Radiology, Korea University Ansan Hospital, Korea University College of Medicine, 123 Jeokgeum-ro, Danwon-gu, Ansan, Gyeonggi-do 15355 Republic of Korea; 4 Department of Radiology, Inha University Hospital and College of Medicine, Inhang-ro 27, Jung-gu, Incheon, 22332 Republic of Korea; 5 Division of Hematology/Oncology, Department of Internal medicine, Korea University Ansan Hospital, Korea University College of Medicine, Ansan, Gyeonggi-do Republic of Korea; 6 Division of Breast and Endocrine Surgery, Department of Surgery, Korea University Ansan Hospital, Korea University College of Medicine, Ansan, Gyeonggi-do Republic of Korea; 7 Department of Radiology, Korea University Anam Hospital, Korea University College of Medicine, Seoul, Republic of Korea; 8 Department of Radiology, Korea University Guro Hospital, Korea University College of Medicine, Seoul, Republic of Korea; 9 Department of Pathology, Korea University Ansan Hospital, Korea University College of Medicine, Ansan, Gyeonggi-do Republic of Korea

**Keywords:** Ultrasonography, Sequence analysis, DNA, Breast neoplasms, Biomarkers, Prospective studies

## Abstract

There are few radiogenomic studies to correlate ultrasound features of breast cancer with genomic changes. We investigated whether vascular ultrasound phenotypes are associated with breast cancer gene profiles for predicting angiogenesis and prognosis. We prospectively correlated quantitative and qualitative features of microvascular ultrasound (vascular index, vessel morphology, distribution, and penetrating vessel) and contrast-enhanced ultrasound (time–intensity curve parameters and enhancement pattern) with genomic characteristics in 31 breast cancers. DNA obtained from breast tumors and normal tissues were analyzed using targeted next-generation sequencing of 105 genes. The single-variant association test was used to identify correlations between vascular ultrasound features and genomic profiles. Chi-square analysis was used to detect single nucleotide polymorphisms (SNPs) associated with ultrasound features by estimating *p* values and odds ratios (ORs). Eight ultrasound features were significantly associated with 9 SNPs (*p* < 0.05). Among them, four ultrasound features were positively associated with 5 SNPs: high vascular index with rs1136201 in *ERBB2* (*p* = 0.04, OR = 7.75); large area under the curve on contrast-enhanced ultrasound with rs35597368 in *PDGFRA* (*p* = 0.04, OR = 4.07); high peak intensity with rs35597368 in *PDGFRA* (*p* = 0.049, OR = 4.05) and rs2305948 in *KDR* (*p* = 0.04, OR = 5.10); and long mean transit time with rs2275237 in *ARNT* (*p* = 0.02, OR = 10.25) and rs755793 in *FGFR2* (*p* = 0.02, OR = 10.25). We identified 198 non-silent SNPs in 71 various cancer-related genes. Vascular ultrasound features can reflect genomic changes associated with angiogenesis and prognosis in breast cancer.

**Supplementary Information:**

The online version contains supplementary material available at 10.1007/s12672-023-00657-8.

## Introduction

Tumor hypoxia is the pathophysiological consequence of the rapid proliferation of tumor cells and relative insufficiency of oxygen supply [1]. To overcome hypoxic stress, hypoxia-inducible factor-1 (HIF-1) is overexpressed in cancer patients, which increases tumor angiogenesis, genetic instability, tissue invasion, and metastasis [1, 2]. Tumor angiogenesis refers to the generation of new vessels from the existing vasculature and is an essential phenomenon for tumor progression in breast cancer [1, 3]. The molecular basis of the angiogenic switch is the increased production of angiogenic factors such as vascular endothelial cell growth factor (VEGF) and the loss of angiogenic inhibitors [3]. To provide better predictors of tumor aggressiveness, it is important to understand the pathophysiological features of breast cancer and the roles of tumor hypoxia and angiogenesis.

In oncology imaging, radiogenomics is a new evolution that relates the imaging properties of cancer to genetic alterations [4, 5]. Radiogenomic investigations may be useful for understanding the heterogeneity of breast cancer and developing noninvasive imaging surrogate markers for predicting outcomes and stratifying patients for more precise therapeutic care [5]. Since the first radiogenomic study by Yamamoto et al. reported that 21 magnetic resonance imaging traits are associated with 71% of the total genes measured in breast cancer patients, most radiogenomic studies of breast cancer have used dynamic contrast-enhanced magnetic resonance imaging [6]. In a more recent radiogenomic study using ultrasound (US) and whole RNA sequencing, 2 B-mode US features and 8 vascular US features were associated with significant upregulation or downregulation of 26 genes relevant to breast cancer in terms of hormone receptor status, angiogenesis, or prognosis [7]. However, few radiogenomic studies have used US and DNA sequencing to investigate breast cancer. Such an approach may provide mutation profiling at the DNA level, which may be useful for exploring the genomic markers associated with US imaging features and improving the prediction and treatment of the disease. The DNA sequencing approach can be used to identify the effects of genomic alterations, such as point mutations, short indels, and structural variations in the cancer genome. Advances in vascular US have facilitated the application of microflow imaging to the evaluation of tumors. Recent investigations have reported that 2 microflow evaluation techniques—superb microvascular imaging (Microvascular US) and contrast-enhanced US (CEUS)—are valuable for distinguishing breast cancer from benign tumors [8]. Although contrast-enhanced magnetic resonance imaging is a good imaging tool for evaluating tumor vascularity as well as morphology, recent CEUS techniques using second-generation contrast agents have an equivalent diagnostic ability for breast cancer but cost less and have easier access [9]. Microvascular US and CEUS can be helpful for predicting histological prognostic factors for breast cancer such as microvessel density, histological grade, negative estrogen receptor expression, and positive human epidermal growth factor receptor 2 (HER2) expression [8, 10].

The purpose of this study is to investigate whether vascular US imaging characteristics observed with Microvascular US and CEUS are associated with certain genomic profiles obtained using DNA sequencing. We hypothesized that vascular US features of breast cancer might be associated with specific genetic alterations, especially those related to angiogenesis or cancer prognosis, as vascular US features correlate with immunohistochemical findings of histological prognostic biomarkers in breast cancer.

## Materials and methods

### Study population

This prospective study was approved by the institutional review board of Korea University Ansan Hospital and conducted in accordance with the ethical principles of the Declaration of Helsinki. Written informed consent was obtained from all patients. From January to October 2016, 98 consecutive patients scheduled to undergo a US-guided core needle biopsy for suspicious breast masses (57 benign and 41 malignant) were included in a prior prospective study to evaluate the usefulness of US vascular imaging (Microvascular US and CEUS) for distinguishing between benign and malignant masses [8]. Of the 41 patients with malignant breast masses, 31 patients (mean age 49.4 years, range 36–76) who underwent breast cancer surgery and provided written informed consent for the use of their gene sequencing data were included in the current study. These 31 patients had been included in another previous study to compare US features with data obtained from whole RNA sequencing [7]. In this study, preoperative vascular US imaging (Microvascular US and CEUS) features and clinicopathological variables for 31 patients were analyzed, and targeted DNA sequencing was performed using next-generation sequencing of breast cancer surgical specimens.

### Technique for vascular US

Vascular US imaging (Microvascular US and CEUS) was performed using an Aplio 500 system (Canon Medical Systems, Tokyo, Japan) with a 5–14 MHz linear transducer by a radiologist (B.K.S) with 18 years of experience in breast imaging. Microvascular US was performed and the plane with the most abundant blood vessels on Microvascular US was saved as a representative image for evaluation. The image parameters for Microvascular US were velocity scale < 3 cm/s, dynamic range 21 dB, and frame rate 27–60 frames/s. CEUS examination was performed using the SonoVue intravenous contrast agent (Bracco, Milan, Italy) after Microvascular US. First, 3.6 mL of the contrast agent was administered, and continuous scanning was performed for 2 min to acquire the video clip for time–intensity curve analysis. After an 8 min wait from the first contrast injection, 1.2 mL of the contrast agent was administered, and the video clip was recorded for 1 min [8, 10–12]. The CEUS image parameters were mechanical index 0.08, frame rate 10 frames/s, gain 80, and dynamic range 65 dB.

### US analysis

Two radiologists (O.H.W. and A.Y.P.) with 12 years and 5 years of experience in breast imaging, respectively), who were blinded to the clinical and histopathological findings, analyzed the following vascular imaging features together and achieved consensus [7, 8]. In case of disagreement, another radiologist (B.K.S.) evaluated the US image until a consensus was reached by three radiologists. Microvascular US was evaluated on static images and CEUS was evaluated on video clips.

For Microvascular US, the vascular index (%, the ratio between the pixels for the Doppler signal and those for the whole lesion) was measured as a quantitative parameter [8, 13, 14]. Vessel morphology (none or simple vs. complex), distribution (none or peripheral only vs. central), and penetrating vessels (absent vs. present) were evaluated as qualitative parameters [8, 14].

For CEUS, the time-intensity curve was evaluated to provide quantitative parameters and the enhancement patterns to provide qualitative parameters [8, 11, 12, 14]. The time-intensity curve parameters included peak intensity (×10^–5^ AU [arbitrary units], the maximum intensity of the time–intensity curve), time to peak (in s, the time needed to reach peak intensity), mean transit time (in s, the time when the intensity was higher than the mean value), slope (×10^–5^ AU/s, the maximum wash-in velocity of the contrast agent), and area under the curve (×10^–5^ AU·s, the integral value of the curve associated with the total blood volume and the sum of the area wash-in and area wash-out). The quantitative parameters were dichotomized by the mean value for the 31 cancers. The enhancement patterns included enhancement degree relative to the surrounding normal parenchyma (iso- or hypoenhancement vs. hyperenhancement), order (centrifugal or diffuse vs. centripetal), margin (circumscribed vs. uncircumscribed), internal homogeneity (homogeneous vs. heterogeneous), penetrating vessels (absent vs. present), and perfusion detection (absent vs. present).

### DNA sequencing analysis

Targeted DNA sequencing was performed for the genomic DNA obtained from the 31 breast cancers and 10 samples of adjacent normal tissues using the Agilent Customized SureDesign Kit 1 (Agilent Technologies, Santa Clara, CA, USA), which includes 105 angiogenesis- and cancer-related genes [15–19] (Table 1 and Supplementary Table 1). To generate the standard exome capture libraries, the Agilent SureSelect Target Enrichment protocol was used with 200 ng of input DNA according to the manufacturer’s instructions. Next-generation sequencing was performed using a HiSeq 2500 system (Illumina, San Diego, CA, USA).

**Table 1:** Composition of customized targeted gene panel

Functions	Gene name
Angiogenesis/hypoxia-related genes	*AKT1, AKT3, ANGPT1, ANGPT2, ARAF, ARNT, EGFR, FGFR1, FGFR2, FGFR3, FGFR4, FIGF, HIF1A, JAK1, JAK2, JAK3, KDR, KIT, MET, NKFBIZ, PDGFB, PDGFRA, PDGFRB, PIGF, SPP1, SRC, TGFB1, VEGF-A, VEGF-C, VHL*
Breast cancer-related genes	*AURKA, AURKB, BARD1, BIRC5, BRCA1, BRCA2, BRIP1, CDH1, CHEK2, CXCL13, ERBB2, ERBB3, ESR1, FoxM1, IGF1R, IGF2, PALB2, PICK3CA, PTTG1, RAD50, RAD51C, RRM2, RUNX1, SPP1, STAT1, STK11, TOP2A, TUBB3, UBE2C*
Cancer-related gene	*ABL1, APC, ATM, BCL2, BRAF, CDK4, CDKN2A, CSF1R, CTNNB1, EPCAM, FBXW7, FOXL2, GNA11, HNF1A, HRAS, IDH1, KRAS, MLH1, MPL, MRE11A, MSH2, MSH3, MSH6, MTOR, MUTHY, MYC, MYCN, NBN, NF1, NOTCH1, NPM1, NRAS, NTRK1, NTRK2, NTRK3, PTEN, RB1, RET, ROS1, RSPO1, RSPO2, SMAD4, SMARCB1, SMO, SYK, TP53, XPO1*

Target gene panel was composed of all coding exons and selected introns of 105 target genes (region size, 308.591 kilobase and average sequencing depth, 1501.8×)

The paired-end sequencing raw data (FASTQ format) were aligned to the human reference genome (hg19) using Burrows–Wheeler aligner-maximum exact matches [20]. Duplicated reads of genomic DNA libraries were discarded using Picard MarkDuplicates (version 2.2.4), and the base quality of deduplicated reads was recalibrated using GATK4 BaseRecalibrator. Somatic mutations were detected using MuTect2 [21] and mutations were examined using GATK4 FilterMutectCalls, FilterAlignmentArtifacts, and FilterByOrientationBias to filter out false-positive calls. ANNOVAR (Annotates VARriation) was used for functional annotation of each variant in coding regions [22]. Germline variants were excluded when the minor allele frequency was ≥ 1% in the Genome Aggregation Database for East Asians (gnomAD, 2.1.1 release) and Korean Variant Archive [23]. To minimize false-positive variants, variants were filtered if they were shown to be present in > 50% of normal samples.

### Statistical analysis

The single-variant association test was performed to evaluate the relationships between US imaging features, clinicopathological variables and genomic profiles. Clinicopathological variables included tumor stage (stage 0 or I vs. II or III), the presence of lymphovascular invasion (absence vs. presence), and pathological nodal staging (0 vs. 1 or 2). Variants were filtered out using the following parameters: the exact test for the Hardy–Weinberg equilibrium (*p* value < 0.001) and single nucleotide polymorphisms (SNPs) call rates < 95%. To detect SNPs associated with US features, chi-square test was used to estimate *p* values and odds ratios (ORs). Analyses were conducted using PLINK [24]. We also performed pathway enrichment analysis using ToppFun in the ToppGene Suite [25] to analyze functionally the genes of interest identified in the 31 breast cancers. The ToppFun program was applied using the default parameters to annotate target genes for gene-ontology molecular function, biological process, cellular component and curated pathway terms, and *p* values were obtained using the probability density function. Pathways with a Bonferroni *q* value ≤ 0.01 were selected as top pathways.

## Results

### Lesion characteristics

Table 2 shows a summary of the vascular US and clinicopathological characteristics of the breast cancers. The tumor size ranged from 7 to 48 mm (mean, 22 mm). The mean values of the quantitative vascular parameters were 16.1% vascular index on Microvascular US, 34.2 × 10^–5^ AU for peak intensity, 6.8 s for time to peak, 14.4 s for mean transit time, 10.2 × 10^–5^ AU/s for slope, and 890.0 × 10^–5^ AU·s for area under the curve on CEUS. Twenty-seven of 31 cancers were invasive ductal carcinomas and 4 were ductal carcinoma in situ. Twenty-three cancers were luminal, 5 were HER2-enriched, and 3 were triple-negative.

**Table 2:** The US and pathological characteristics of 31 breast cancer

Characteristics	Number of cancer
Tumor size	
≤ 20 mm	17 (54.8)
> 20 mm	14 (45.2)
Microvascular US	
Vascular index	
< 16.1%	19 (61.3)
≥ 16.1%	12 (38.7)
Vessel morphology	
None or simple	6 (19.4)
Complex	25 (80.6)
Vessel distribution	
None or peripheral	2 (6.5)
Central	29 (93.5)
Penetrating vessel	
Absent	6 (19.4)
Present	25 (80.6)
CEUS	
Peak intensity	
< 34.2 × 10^−5^ AU	18 (58.1)
≥ 34.2 × 10^−5^ AU	13 (41.9)
Time to peak	
> 6.8 s	13 (41.9)
≤ 6.8 s	18 (58.1)
Mean transit time	
< 14.4 s	21 (67.7)
≥ 14.4 s	10 (32.3)
Slope	
< 10.2 × 10^−5^ AU /s	22 (71.0)
≥ 10.2 × 10^−5^ AU /s	9 (29.0)
Area under the curve	
< 890.0 × 10^−5^ AU·s	21 (67.7)
≥ 890.0 × 10^−5^ AU·s	10 (32.3)
Enhancement degree	
Iso or hypo-enhancement	2 (6.5)
Hyper-enhancement	29 (93.6)
Enhancement order	
Centrifugal or diffuse	9 (29.0)
Centripetal	22 (71.0)
Enhancement margin	
Circumscribed	13 (41.9)
Uncircumscribed	18 (58.1)
Internal homogeneity	
Homogeneous	17 (54.8)
Heterogeneous	14 (45.2)
Penetrating vessel	
Absent	11(35.5)
Present	20 (64.5)
Perfusion defect	
Absent	20 (64.5)
Present	11 (35.5)
Clinicopathological findings	
Tumor type	
Invasive ductal carcinoma	27 (87.1)
Ductal carcinoma in situ	4 (12.9)
Immunohistochemical results	
ER positivity	21 (67.7)
PR positivity	22 (71.0)
HER2 positivity	8 (25.8)
Lymph node metastasis	
Absent	22 (71.0)
Present	9 (29.0)
Lymphovascular invasion	
Absent	21 (67.7)
Present	10 (33.3)
Stage	
0	4 (12.9)
I	11 (35.5)
II	13 (41.9)
III	3 (9.7)

Data are the number of cancers with percentages in parentheses

*US* ultrasound, *CEUS* contrast-enhanced ultrasound, *AU* arbitrary units, *ER* estrogen receptor, *PR* progesterone receptor, *HER2* human epidermal growth factor receptor 2

Vascular parameters were analyzed according to the tumor size (≤ 20 mm vs. > 20 mm) was analyzed. The peak intensity and area under the curve on CEUS were higher in tumors measuring > 20 mm than in those ≤ 20 mm (42.5 × 10^–5^ AU vs. 27.3 × 10^–5^ AU, *p* = 0.04; 1120.4 × 10^–5^ AU·s vs. 718.0 × 10^–5^ AU·s, *p* = 0.04), respectively. None of the other parameters differed significantly according to tumor size.

### Genomic profiling of breast cancer

**Fig. 1:**
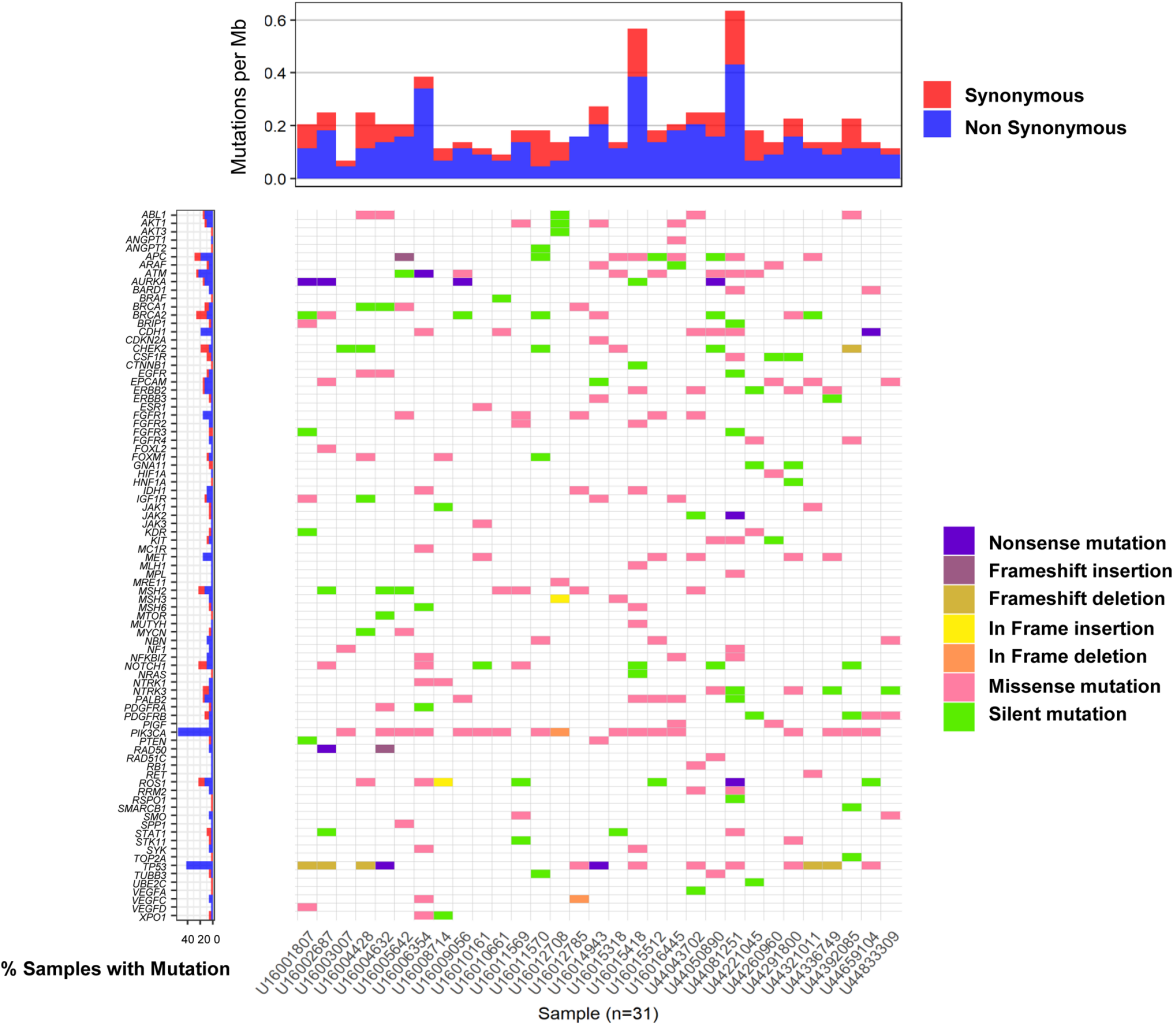
Genomic landscape represented by angiogenesis and cancer-related genes in 31 breast cancer patients. A total of 198 non-silent (nonsynonymous) mutations in 71 genes were identified. The x column indicates 31 cancer patients, and the y column indicates the 71 genes with 198 non-silent mutations (small color bars except for green). *PIK3CA* showed the most frequent mutations in breast cancer genomes (16/31, 51.6%). Other recurrent non-silent mutations (≥ 3 cases) were found in 24 breast cancer- or cancer-related genes including *TP53* (13 cases), *ATM* (7 cases), *CDH1* (7 cases), *APC* (6 cases), *ERBB2* (5 cases), *FGFR1* (5 cases), and *MET* (5 cases)

We identified a total of 198 nonsilent mutations that have observable effects on the phenotype, in 71 genes. *PIK3CA* showed the most frequent mutations in breast cancer genomes, 22 nonsilent mutations in 16 breast cancers (51.6%). Other recurrent nonsilent mutations (≥ 3 cases) were found in 24 breast cancer- or cancer-related genes including *TP53* (13 cases), *ATM* (7 cases), *CDH1* (7 cases), *APC* (6 cases), *ERBB2* (5 cases), *FGFR1* (5 cases), and *MET* (5 cases). Figure 1 shows the genomic landscape represented by 71 angiogenesis- and cancer-related genes in 31 breast cancer patients. Pathway enrichment analysis of the 71 genes showed that these patients had significant enrichment of various cancer-related functions such as regulation of cell death and regulation of cell population proliferation (*q* value ≤ 0.01). The results of this analysis also identified genes related to angiogenesis, such as signaling by platelet-derived growth factor (PDGF) (*q* value = 2.43 × 10^–15^) and VEGF (*q* value = 4.22 × 10^–15^). Supplementary Table 2 shows the top 100 gene sets and pathways from pathway enrichment analysis using 71 genes.

### Associations between US features and genomic profiles

**Fig. 2:**
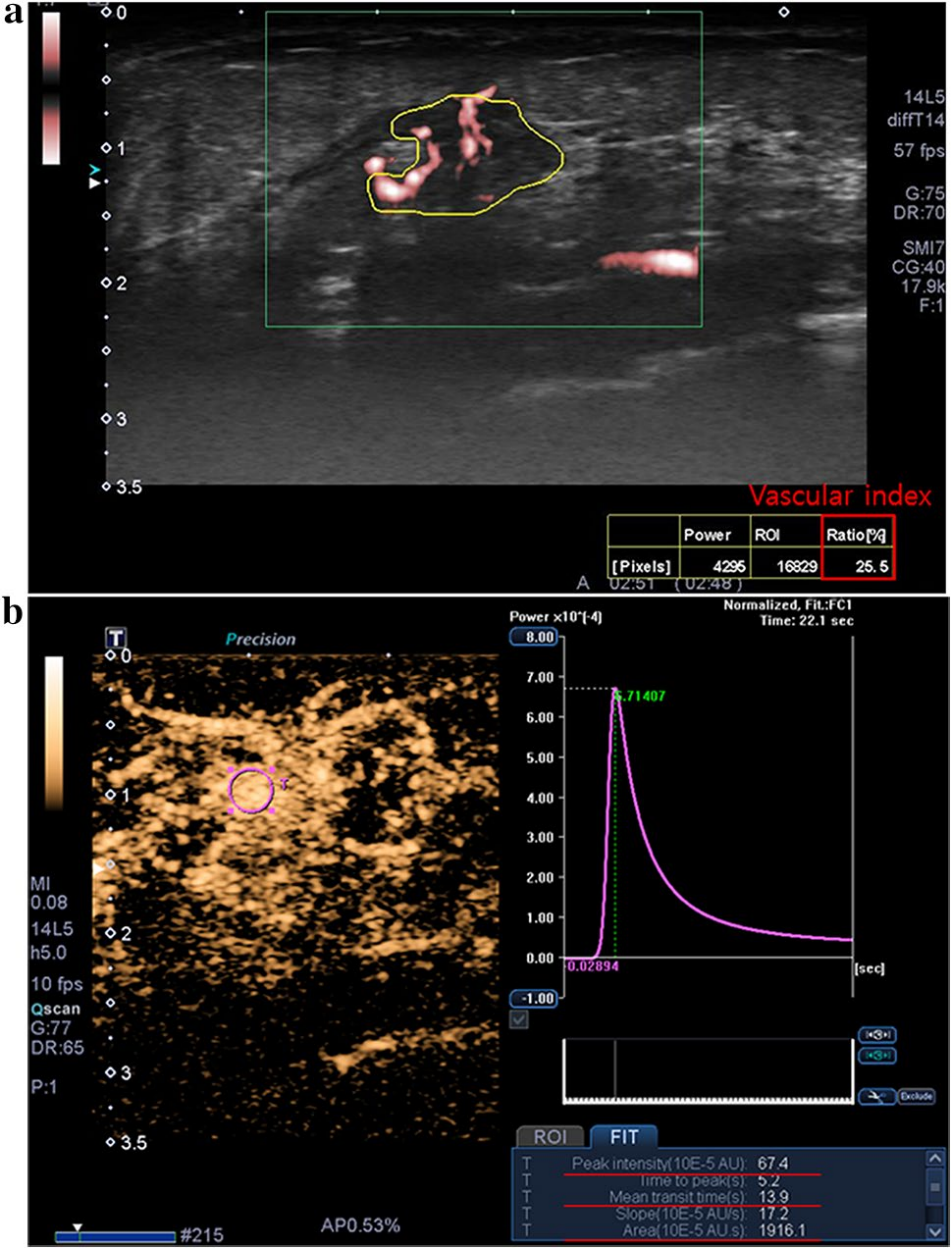
A 53-year-old female with invasive ductal carcinoma. **a** A microvascular US image shows an increased vascular index, 25.6% (above the mean value of 16.1%) (red box). **b** A contrast-enhanced US image shows high peak intensity, 67.4 × 10^−5^ AU (above the mean value of 34.2 × 10^−5^ AU), and area under the curve, 1916.1 × 10^−5^ AU·s (above the mean value of 890.0 × 10^−5^ AU·s), and low mean transit time, 13.9 s (below the mean value of 14.4 s). DNA sequencing results for the cancer showed the presence of SNP rs1136201 in *ERBB2* gene, SNP rs2305948 in *KDR* gene, and SNP rs35597368 in *PDGFRA* gene. SNP, single nucleotide polymorphism

**Fig. 3:**
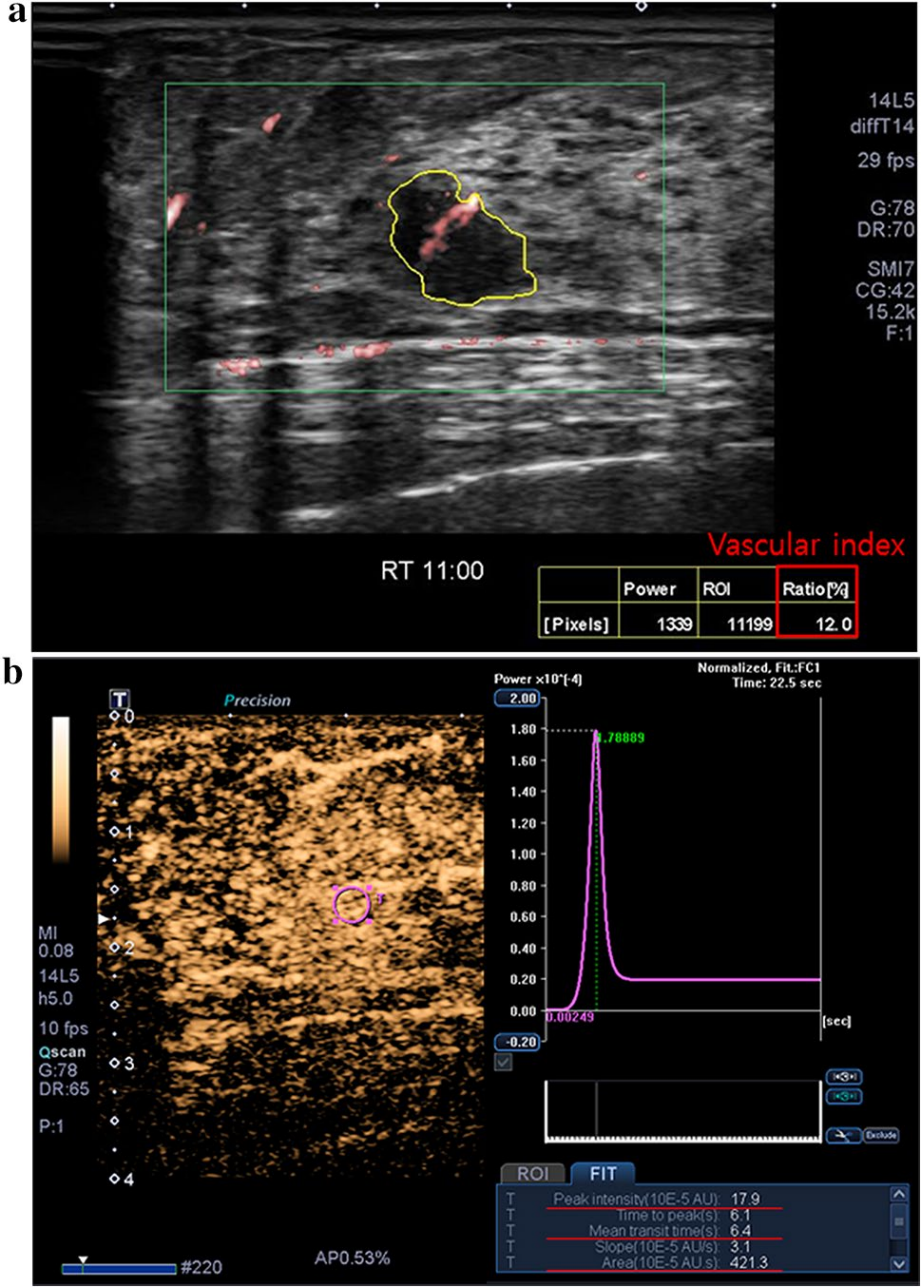
A 49-year-old female with invasive ductal carcinoma. **a** A microvascular US image shows a low vascular index, 12.0% (below the mean value of 16.1%) (red box). **b** A contrast-enhanced US image shows low peak intensity, 17.9 × 10^− 5^ AU (below the mean value of 34.2 × 10^−5^ AU), mean transit time, 6.4 s (below the mean value of 14.4 s), and area under the curve, 421.3 × 10^− 5^ AU·s (below the mean value of 890.0 × 10^− 5^ AU·s). DNA sequencing results for the cancer showed the absence of SNP rs1136201 in *ERBB2*, rs35597368 in *PDGFRA*, rs2305948 in *KDR*, rs2275237 in *ARNT*, and rs755793 in *FGFR2*. SNP, single nucleotide polymorphism

**Fig. 4:**
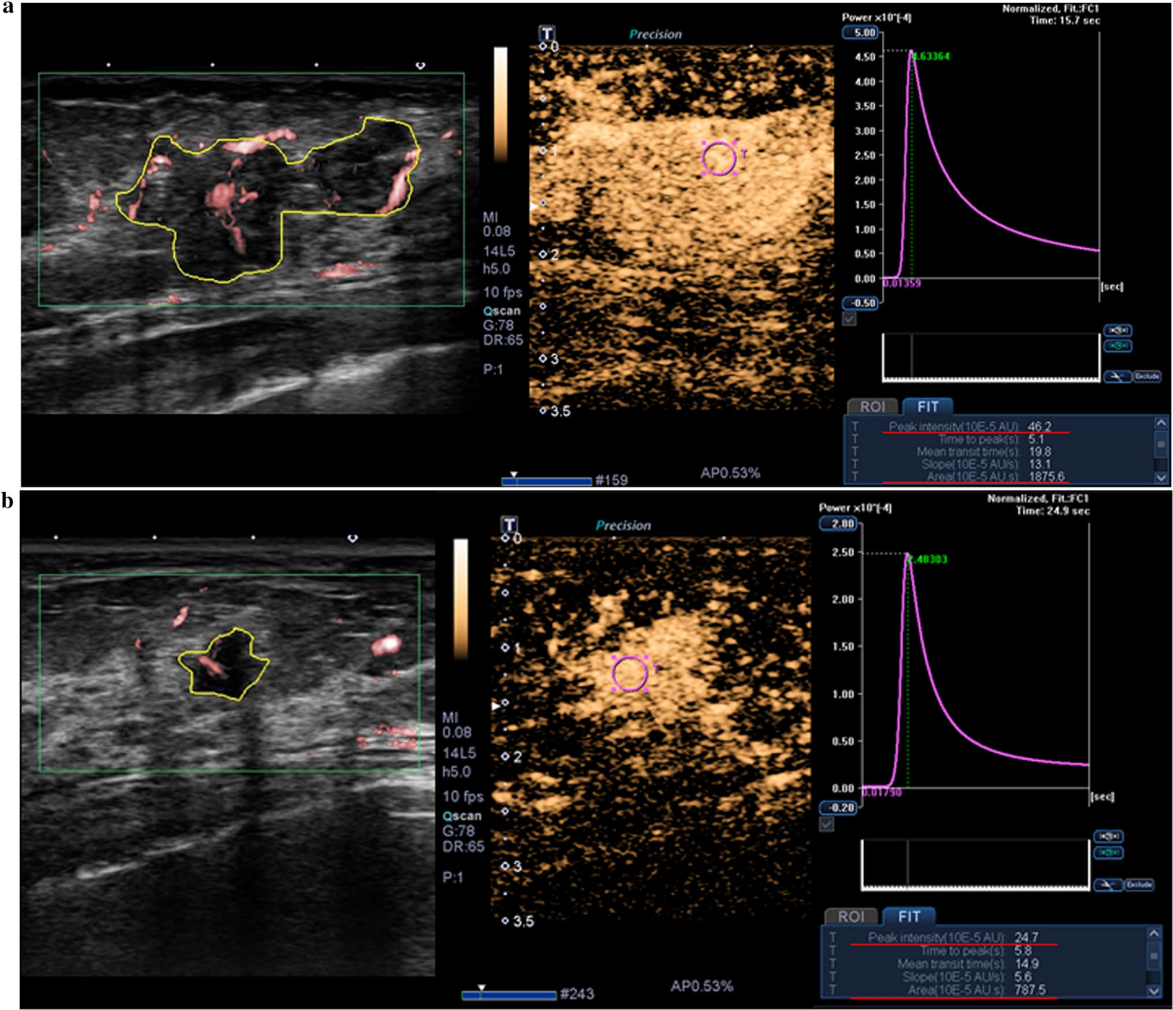
Comparison of vascular US parameters according to the tumor size (≤ 20 mm vs. > 20 mm). **a** Microvascular US and contrast-enhanced US images of a 35 mm-sized invasive ductal carcinoma in a 47-year-old female show increased peak intensity, 46.2 × 10^−5^ AU (above the mean value of 34.2 × 10^−5^ AU), and area under the curve, 1875.6 × 10^−5^ AU·s (above the mean value of 890.0 × 10^−5^ AU·s). DNA sequencing results for the cancer showed the presence of rs35597368 in *PDGFRA*. **b** Microvascular US and contrast-enhanced US images of a 14 mm-sized invasive ductal carcinoma in a 62-year-old female show low peak intensity, 24.7 × 10^−5^ AU (below the mean value of 34.2 × 10^−5^ AU), and area under the curve, 787.5 × 10^−5^ AU·s (below the mean value of 890.0 × 10^−5^ AU·s). DNA sequencing results for the cancer showed the absence of rs35597368 in *PDGFRA* and rs2305948 in *KDR*. SNP, single nucleotide polymorphism

We investigated the associations between vascular US features and SNPs in the breast cancer samples. Nine SNPs were significantly associated with 8 vascular US features (*p* < 0.05) and 3 SNPs (rs2305948 [C>T] in *KDR*, rs35597368 [T>C] in *PDGFRA*, and rs152451 [T>C] in *PALB2*) were associated with two different vascular US features (Table 3). Using RNA sequencing data [7], we evaluated the expression levels of transcripts for these 9 SNPs and found that transcripts associated with all 9 SNPs showed transcript-level expression. Among the 12 associations between vascular US imaging features and genomic profiles observed, 4 vascular US features showed positive associations with 5 SNPs (OR > 1) as follows: high vascular index on Microvascular US with rs1136201 (A>G) in *ERBB2* (*p* = 0.04, OR = 7.75) (Fig. 2); large area under the time-intensity curve on CEUS with rs35597368 (T>C) in *PDGFRA* (*p* = 0.04, OR = 4.07); high peak intensity with rs35597368 (T>C) in *PDGFRA* (*p* = 0.049, OR = 4.05) and rs2305948 (C>T) in *KDR* (*p* = 0.04, OR = 5.10) (Fig. 3); and long mean transit time with rs2275237 (G>A) in *ARNT* (*p* = 0.02, OR = 10.25) and rs755793 (A>G) in *FGFR2* (*p* = 0.02, OR = 10.25). Figure 2 shows an invasive ductal carcinoma with increased vascular index on Microvascular US, increased peak intensity and area under the curve on CEUS, and the presence of corresponding SNPs. Figure 3 shows an invasive ductal carcinoma with low vascular index on Microvascular US, low peak intensity, mean transit time, and area under the curve on CEUS, as well as the absence of corresponding SNPs. Figure 4 shows the comparison of peak intensity and area under the curve on CEUS as well as the status of corresponding SNPs according to the tumor size. Two vascular US features—vessel distribution on Microvascular US and enhancement degree on CEUS—were excluded from the analyses because data were skewed to one group between the two (Table 2).

**Table 3:** Association between vascular US imaging features and genomic profiles

US Features	Chromosome:position^a^	SNP	Gene	Alleles	*p* value	Odds ratio
Microvascular US						
Vascular index	chr17:37879588	rs1136201	*ERBB2*	A>G	0.04	7.75
Vessel morphology	chr8:38287238	rs140382957	*FGFR1*	G>A	0.004	0.06
	chr10:43610119	rs1799939	*RET*	G>A	0.046	0.19
Penetrating vessel	chr4:55979558	rs2305948	*KDR*	C>T	< 0.001	0.09
	chr17:41244982	rs80356892	*BRCA1*	A>G	0.03	0.10
CEUS						
Peak intensity	chr4:55139771	rs35597368	*PDGFRA*	T>C	0.049	4.05
	chr4:55979558	rs2305948	*KDR*	C>T	0.04	5.10
Mean transit time	chr1:150785811	rs2275237	*ARNT*	G>A	0.02	10.25
	chr10:123310871	rs755793	*FGFR2*	A>G	0.02	10.25
Area under the curve	chr4:55139771	rs35597368	*PDGFRA*	T>C	0.04	4.07
Enhancement order	chr16:23646191	rs152451	*PALB2*	T>C	0.04	0.26
Enhancement margin	chr16:23646191	rs152451	*PALB2*	T>C	0.003	0.11

*CEUS* contrast-enhanced ultrasound, *SNP* single nucleotide polymorphism

^a^Chromosome position (bp) based on NCBI (National Center for Biotechnology Information) Human Genome Build 37

When we evaluated the correlations between genetic alternations and clinicopathological findings, rs3739390 in *ANGPT1* was associated with the presence of lymph node metastasis (*p* = 0.053). However, none of the vascular US feature evaluated in this study was related to this SNP. Tumor stage and lymphovascular invasion were not associated with any specific SNPs.

## Discussion

We hypothesized that vascular US imaging features of breast cancer would be associated with certain genetic alterations within angiogenesis- or breast cancer-related genes. In this study, we found that 4 quantitative vascular US features—the vascular index on Microvascular US, area under the time-intensity curve, peak intensity, and mean transit time on CEUS—were significantly associated with 5 SNPs in relation to angiogenesis. In addition, the genes that frequently showed recurrent nonsilent mutations in our study are consistent with the top breast cancer genes with driver mutations, *PIK3CA*, *TP53*, *CDH1*, *APC*, *ERBB2*, and *FGFR1* [26].

Breast cancers with an increased vascular index on Microvascular US about 8 times more frequently showed the SNP rs1136201 in *ERBB2* compared with those with a lower vascular index. *ERBB*, commonly referred to as *HER2*, is overexpressed in 20% of breast cancer and is considered to be a marker of aggressive disease [27]. HER2-positive cancer has been reported to be related to higher levels of angiogenesis via the close relationship between HER2 signaling and angiogenesis at different levels of molecular crosstalk [27]. Radiology investigations have shown that HER2-positive breast cancers are associated with a higher degree of tumor vascularity on US microvascular imaging [28, 29]. The vascular index is an objective US parameter for assessing the degree of tumor vascularity [8]. Therefore, in this study, the SNP in *ERBB2* may be an important genetic alteration that promotes tumor angiogenesis and is manifested as an increased vascular index on Microvascular US. In particular, rs1136201 (A>G) in *ERBB2*, also referred to as the *HER2* Ile655Val polymorphism, has been investigated as a susceptibility factor for breast cancer because Val-expressing cells exhibit increased proliferation and reduced apoptosis in vitro [30]. Han et al. reported that patients with HER2-positive cancers with the Val variant have a shorter disease-free survival, but a greater sensitivity to trastuzumab treatment [31]. Therefore, HER2-positive breast cancer showing an increased vascular index on Microvascular US may be associated with rs1136201 in *ERBB2* and may be expected to have a more favorable response to targeted therapy.

On the time-intensity curve of CEUS, breast cancers with increased peak intensity had about 5 times the frequency of the SNP rs2305948 in *KDR* compared with those with low peak intensity. In addition, this SNP was predicted to be functioning in the SNP annotation test. Peak intensity reflects the fractional blood volume and flow passing through the region of interest and correlates positively with microvessel density in breast cancer, which suggests that peak intensity can predict tumor angiogenesis [8, 9]. *KDR* is the human gene encoding VEGF receptor 2, which plays a major role in mediating VEGF-induced responses such as endothelial cell development and the direct regulation of tumor angiogenesis [32]. *KDR* expression has been identified as an independent predictor of a complete response to neoadjuvant chemotherapy in triple-negative breast cancer patients, and rs2305948 in *KDR* also tended to be associated with the treatment response [33]. Therefore, the increase in peak intensity in breast cancer on CEUS may be related to rs2305948 in *KDR* and may be a potential indicator of tumor angiogenesis and the response to chemotherapy especially in triple-negative cancer patients.

Cancers with a larger area under the curve and peak intensity on CEUS were associated with about 4 times the frequency of mutations at rs35597368 in *PDGFRA* compared with those with a lower area under the curve and peak intensity. PDGF receptor A is a tyrosine kinase receptor for PDGF ligands. Overexpression or mutation of *PDGFR*A causes the dysregulation of PDGF signaling and promotes angiogenesis, cell migration, and invasion [34]. High expression of *PDGFRA* in breast cancer cells is related to poor histological features such as lymph node metastasis, HER2 positivity, high histological grade, and triple-negative cancer subtype [34]. Therefore, an increased area under the curve and peak intensity on CEUS might reflect increased tumor angiogenesis and perfusion related to mutations in *PDGFRA*.

Breast cancers with a longer mean transit time in the time-intensity curve on CEUS were 10 times more likely to be associated with the SNPs-rs2275237 in *ARNT* and rs755793 in *FGFR2* compared with those with a shorter mean transit time. A long mean transit time reflects a long circulation time of the contrast medium inside the lesion and can imply a high degree of tumor angiogenesis [9, 35]. Human *ARNT* encodes HIF-1β, which produces HIF-1 via heterodimeric binding with HIF-1α. In the absence of oxygen, HIF-1 increases the transcription of various genes for angiogenesis, glucose metabolism, and cell proliferation and migration, which activate an adaptive response to tumor hypoxia [36]. Therefore, *ARNT* mutations may play a role in HIF-1 complex activation and promote tumor angiogenesis, which may appear as an increase in the mean transit time on CEUS. Fibroblast growth factor receptor 2 (FGFR2) protein, which is encoded by *FGFR2*, triggers an FGFR2-mediated signaling pathway that plays a role in cell proliferation, differentiation, and angiogenesis [37]. Therefore, we suggest that *FGFR2* polymorphism may affect the FGFR2-mediated signaling pathway and promote tumor angiogenesis; if so, this may appear as an increased mean transit time on CEUS.

Our study has some limitations. First, the small study population and the absence of validation cohort investigation may imply that our study is underpowered. We found a correlation between genetic alterations and clinicopathological findings, such as ANGPT1 mutations associated with lymph node metastasis, but because of the small study population, an integrated association among genetic alterations, clinicopathological findings, and vascular US features could not be evaluated. However, in this study, the false-positive results and germline mutations were filtered out using normal tissue samples and East Asian and Korean genome databases. In addition, the genes frequently representing recurrent nonsilent mutations in this study are consistent with the landscape of driver mutations in 100 breast cancers reported in a previous study [26]. Further investigations using large populations are needed in the near future to validate our results and demonstrate an integrative relationship between imaging features, clinicopathological findings, and genetic data. Furthermore, the occurrence of certain phenotypic changes in tumor cells may not be explained by a single SNP because multiple genetic events and additional biological reactions may be required. Therefore, to better understand the associations between SNPs and breast imaging features in our study, additional in vitro and in vivo experiments should be performed. Second, the vascular US features evaluated in this study were not based on a quantitative radiomics approach. We used the built-in software commonly used in US equipment for quantitative evaluation of tumor vessels during US examination. We aimed to evaluate the applicability of radiogenomic research using US in daily clinical practice. Finally, the effect of tumor size on vascular US parameters and genetic changes was not considered in this study, although the tumors showed a wide range of size from 7 to 48 mm. Future studies should include a subgroup analysis by tumor size in a larger study population. In conclusion, quantitative vascular US features including the vascular index on Microvascular US and the peak intensity, mean transit time, and area under the time-intensity curve on CEUS were associated with frequent mutations in *ERBB2*, *KDR*, *PDGFRA*, *ARNT*, and *FGFR2. *We speculate that vascular US examination may indicate the altered vascular microenvironment of breast cancer probably resulting from these genetic changes, and may provide surrogate markers that could be useful when making decisions about treatment and predicting the prognosis of breast cancer. Further large-scale investigations using whole-exome sequencing and clinicopathological validation are needed to verify the results.

## Supplementary Information

Supplementary file 1

## Data Availability

Please contact authors for data requests.

## Abbreviations

HIF-1: Hypoxia-inducible factor-1
VEGF: Vascular endothelial cell growth factor
US: Ultrasound
Microvascular US: Superb microvascular imaging
CEUS: Contrast-enhanced ultrasound
HER2: Human epidermal growth factor receptor 2
AU: Arbitrary units
SNP: Single nucleotide polymorphisms
OR: Odds ratios
PDGF: Platelet-derived growth factor
