# Supplementary material for: Association between vascular ultrasound features and DNA sequencing in breast cancer: a preliminary study
Source: Discov Oncol. 2023 Apr 30;14:52. doi: 10.1007/s12672-023-00657-8 (PMC10149538; doi:10.1007/s12672-023-00657-8)
Supplement: Supplementary file 1 [file 12672_2023_657_MOESM1_ESM.docx]

| **Supplementary Table 1** Description of targeted DNA sequencing data in breast cancer genomes | | | | |  |
| --- | --- | --- | --- | --- | --- |
| **Sample ID** | **Sequencing reads** | **Mapped reads** | **Mapped reads in exon (%)** | **Coverage (mean)^*^** | **Mean mapping quality** |
| U16001807 | 15,877,522 | 6,612,570 | 54.60 | 1586.00 | 59.60 |
| U16002687 | 16,054,206 | 6,003,580 | 53.70 | 1438.20 | 59.62 |
| U16003007 | 14,532,930 | 5,682,280 | 54.30 | 1319.50 | 59.64 |
| U16004428 | 14,917,184 | 6,542,410 | 54.40 | 1562.10 | 59.54 |
| U16004632 | 15,577,932 | 4,303,344 | 44.60 | 1018.30 | 59.65 |
| U16005642 | 15,011,486 | 5,519,509 | 52.40 | 1315.80 | 59.59 |
| U16006354 | 15,824,902 | 6,343,997 | 50.70 | 1494.20 | 59.52 |
| U16008714 | 16,099,280 | 4,727,011 | 46.00 | 1109.70 | 59.63 |
| U16009056 | 14,549,782 | 5,956,291 | 50.80 | 1407.80 | 59.56 |
| U16010161 | 15,148,136 | 6,113,903 | 50.00 | 1452.40 | 59.56 |
| U16010661 | 14,633,786 | 6,035,786 | 54.30 | 1430.30 | 59.60 |
| U16011569 | 15,565,152 | 6,136,348 | 53.10 | 1468.50 | 59.57 |
| U16011570 | 15,172,896 | 6,693,792 | 54.80 | 1600.80 | 59.56 |
| U16012708 | 15,638,392 | 5,722,624 | 52.00 | 1373.80 | 59.58 |
| U16012785 | 15,179,502 | 5,594,427 | 53.80 | 1329.00 | 59.57 |
| U16014943 | 14,580,748 | 6,502,521 | 54.00 | 1543.00 | 59.55 |
| U16015318 | 16,005,904 | 6,629,116 | 52.80 | 1568.80 | 59.52 |
| U16015418 | 15,665,018 | 6,570,367 | 53.90 | 1550.90 | 59.52 |
| U16015512 | 16,252,594 | 5,531,306 | 48.20 | 1300.40 | 59.59 |
| U16016445 | 17,220,484 | 6,814,656 | 52.20 | 1609.30 | 59.51 |
| U44043702 | 14,959,850 | 7,469,465 | 59.20 | 1750.90 | 59.54 |
| U44050890 | 13,487,846 | 7,255,564 | 62.40 | 1703.30 | 59.56 |
| U44081251 | 13,277,050 | 6,486,926 | 62.00 | 1537.00 | 59.57 |
| U44221045 | 14,679,282 | 7,151,728 | 54.50 | 1674.00 | 59.58 |
| U44260960 | 15,702,320 | 7,417,118 | 58.40 | 1761.90 | 59.57 |
| U44291800 | 13,568,410 | 6,807,308 | 60.90 | 1615.00 | 59.59 |
| U44321011 | 12,579,590 | 6,338,561 | 56.10 | 1486.60 | 59.38 |
| U44336749 | 12,831,790 | 5,730,896 | 47.30 | 1339.60 | 59.33 |
| U44392085 | 16,998,486 | 7,761,810 | 50.60 | 1812.90 | 59.53 |
| U44659104 | 14,559,280 | 7,313,392 | 62.10 | 1732.10 | 59.58 |
| U44833309 | 13,858,120 | 7,067,746 | 57.00 | 1663.30 | 59.58 |

^*^The mean coverage was calculated onto the targeted regions (Agilent Customized SureDesign Kit 1)

**Supplementary Table 2** Top 100 gene sets and pathways from pathway enrichment analysis using 71 genes

| Name | *q* value^*^ | Input genes |
| --- | --- | --- |
| Pathways in cancer | 1.87x10^-28^ | *VEGFC,JAK1,CDKN2A,MET,MSH6,ERBB2,HIF1A,IGF1R,ABL1,CSF1R,BRCA2,EGFR,PDGFRA,RB1,PDGFRB,PIK3CA,NTRK1,APC,MLH1,AKT1,SMO,FGFR1,MSH2,MSH3,FGFR2,RET,PTEN, VEGFD,CDH1,KIT,ARAF,STAT1,TP53* |
| Protein autophosphorylation | 8.35x10^-28^ | *VEGFC,JAK2,AURKA,MET,MRE11,STK11,ERBB2,IGF1R,ABL1,CSF1R,EGFR,PDGFRA,PDGFRBNTRK1,CHEK2,SYK,NBN,AKT1,KDR,ROS1,FGFR1,FGFR2,ATM,FGFR4,KIT,RAD50* |
| Regulation of programmed cell death | 2.12x10^-27^ | *MPL,CDKN2A,JAK2,AURKA,JAK3,MET,MRE11,ERBB2,ERBB3,HIF1A,IGF1R,ABL1,NF1,ANGPT1,CSF1R,FOXL2,BRCA1,EGFR,RB1,PDGFRB,SPP1,PIK3CA,NTRK1,ESR1,NTRK3,CHEK2,SYK, APC,BARD1,AKT1,KDR,SMO,FGFR1,MSH2,FGFR2,ATM,RET,PTEN,CDH1,KIT,EPCAM,ARAF, PALB2,NOTCH1,STAT1,TP53* |
| Regulation of cell death | 4.73x10^-27^ | *MPL,CDKN2A,JAK2,MYCN,AURKA,JAK3,MET,MRE11,ERBB2,ERBB3,HIF1A,IGF1R,ABL1,NF1,ANGPT1,CSF1R,FOXL2,BRCA1,EGFR,RB1,PDGFRB,SPP1,PIK3CA,NTRK1,ESR1,NTRK3, CHEK2,SYK,APC,BARD1,AKT1,KDR,SMO,FGFR1,MSH2,FGFR2,ATM,RET,PTEN,CDH1,KIT, EPCAM,ARAF,PALB2,NOTCH1,STAT1,TP53* |
| Regulation of apoptotic process | 3.20x10^-25^ | *MPL,CDKN2A,JAK2,AURKA,JAK3,MRE11,ERBB2,ERBB3,HIF1A,IGF1R,ABL1,NF1,ANGPT1, CSF1R,FOXL2,BRCA1,EGFR,RB1,PDGFRB,SPP1,PIK3CA,NTRK1,ESR1,NTRK3,CHEK2,SYK, APC,BARD1,AKT1,KDR,SMO,FGFR1,MSH2,FGFR2,ATM,RET,PTEN,CDH1,EPCAM,ARAF, PALB2,NOTCH1,STAT1,TP53* |
| Regulation of cell population proliferation | 4.9 x10^-25^ | *MPL,VEGFC,FOXM1,CDKN2A,JAK2,MYCN,JAK3,STK11,ERBB2,ERBB3,HIF1A,BRIP1,IGF1R, ABL1,NF1,ANGPT1,CSF1R,BRCA1,BRCA2,EGFR,PDGFRA,RB1,PDGFRB,PIK3CA,NTRK1,ESR1,NTRK3,SYK,APC,NBN,AKT1,KDR,SMO,FGFR1,FGFR2,ATM,FGFR4,PTEN,VEGFD,CDH1,KIT,EPCAM,NOTCH1,STAT1,TP53* |
| Protein kinase activity | 2.00x10^-24^ | *VEGFC,JAK1,CDKN2A,JAK2,AURKA,JAK3,MET,MRE11,STK11,ERBB2,ERBB3,IGF1R,ABL1, NF1,ANGPT1,CSF1R,EGFR,PDGFRA,RB1,PDGFRB,PIK3CA,NTRK1,NTRK3,CHEK2,SYK,APC,NBN,AKT1,KDR,ROS1,FGFR1,FGFR2,ATM,FGFR4,RET,PTEN,KIT,ARAF,RAD50* |
| Regulation of phosphorylation | 5.58x10^-24^ | *VEGFC,FOXM1,CDKN2A,JAK2,MET,MRE11,STK11,ERBB2,ERBB3,HIF1A,IGF1R,ABL1,NF1, ANGPT1,CSF1R,EGFR,PDGFRA,RB1,PDGFRB,PIK3CA,NTRK1,ESR1,NTRK3,CHEK2,SYK,APC,BARD1,NBN,AKT1,KDR,ROS1,FGFR1,FGFR2,ATM,FGFR4,RET,PTEN,VEGFD,KIT,ARAF, NOTCH1,TP53,RAD50* |
| Kinase activity | 2.22x10^-23^ | *VEGFC,JAK1,CDKN2A,JAK2,AURKA,JAK3,MET,MRE11,STK11,ERBB2,ERBB3,HIF1A,IGF1R, ABL1,NF1,ANGPT1,CSF1R,EGFR,PDGFRA,RB1,PDGFRB,PIK3CA,NTRK1,NTRK3,CHEK2, SYK,APC,NBN,AKT1,KDR,ROS1,FGFR1,FGFR2,ATM,FGFR4,RET,PTEN,KIT,ARAF,TP53,RAD50* |
| Regulation of protein phosphorylation | 3.70x10^-23^ | *VEGFC,FOXM1,CDKN2A,JAK2,MET,MRE11,STK11,ERBB2,ERBB3,IGF1R,ABL1,NF1,ANGPT1, CSF1R,EGFR,PDGFRA,RB1,PDGFRB,PIK3CA,NTRK1,ESR1,NTRK3,CHEK2,SYK,APC,NBN,AKT1,KDR,ROS1,FGFR1,FGFR2,ATM,FGFR4,RET,PTEN,VEGFD,KIT,ARAF,NOTCH1,TP53,RAD50* |
| Regulation of phosphate metabolic process | 3.76x10^-23^ | *VEGFC,FOXM1,CDKN2A,JAK2,MET,MRE11,STK11,ERBB2,ERBB3,HIF1A,IGF1R,ABL1,NF1, ANGPT1,CSF1R,EGFR,PDGFRA,RB1,PDGFRB,PIK3CA,NTRK1,ESR1,NTRK3,CHEK2,SYK,APC,BARD1,NBN,AKT1,KDR,ROS1,FGFR1,FGFR2,ATM,FGFR4,IDH1,RET,PTEN,VEGFD,KIT,ARAF,NOTCH1,TP53,RAD50* |
| Regulation of phosphorus metabolic process | 3.84x10^-23^ | *VEGFC,FOXM1,CDKN2A,JAK2,MET,MRE11,STK11,ERBB2,ERBB3,HIF1A,IGF1R,ABL1,NF1, ANGPT1,CSF1R,EGFR,PDGFRA,RB1,PDGFRB,PIK3CA,NTRK1,ESR1,NTRK3,CHEK2,SYK,APC,BARD1,NBN,AKT1,KDR,ROS1,FGFR1,FGFR2,ATM,FGFR4,IDH1,RET,PTEN,VEGFD,KIT,ARAF,NOTCH1,TP53,RAD50* |
| Phosphotransferase activity, alcohol group as acceptor | 6.48x10^-23^ | *VEGFC,JAK1,CDKN2A,JAK2,AURKA,JAK3,MET,MRE11,STK11,ERBB2,ERBB3,IGF1R,ABL1, NF1,ANGPT1,CSF1R,EGFR,PDGFRA,RB1,PDGFRB,PIK3CA,NTRK1,NTRK3,CHEK2,SYK,APC,NBN,AKT1,KDR,ROS1,FGFR1,FGFR2,ATM,FGFR4,RET,PTEN,KIT,ARAF,RAD50* |
| Protein tyrosine kinase activity | 1.37x10^-22^ | *JAK1,JAK2,JAK3,MET,ERBB2,ERBB3,IGF1R,ABL1,CSF1R,EGFR,PDGFRA,PDGFRB,NTRK1, NTRK3,SYK,KDR,ROS1,FGFR1,FGFR2,FGFR4,RET,KIT* |
| Peptidyl-tyrosine phosphorylation | 3.11x10^-22^ | *VEGFC,JAK1,JAK2,JAK3,MET,STK11,ERBB2,ERBB3,IGF1R,ABL1,ANGPT1,CSF1R,EGFR, PDGFRA,PDGFRB,NTRK1,NTRK3,SYK,KDR,ROS1,FGFR1,FGFR2,FGFR4,RET,KIT,TP53* |
| transferase activity, Transferring phosphorus-containing groups | 3.26x10^-22^ | *VEGFC,JAK1,CDKN2A,JAK2,AURKA,JAK3,MET,MRE11,STK11,ERBB2,ERBB3,HIF1A,IGF1R, ABL1,NF1,ANGPT1,CSF1R,PIGF,EGFR,PDGFRA,RB1,PDGFRB,PIK3CA,NTRK1,NTRK3,CHEK2SYK,APC,NBN,AKT1,KDR,ROS1,FGFR1,FGFR2,ATM,FGFR4,RET,PTEN,KIT,ARAF,TP53,RAD50* |
| Peptidyl-tyrosine modification | 3.74x10^-22^ | *VEGFC,JAK1,JAK2,JAK3,MET,STK11,ERBB2,ERBB3,IGF1R,ABL1,ANGPT1,CSF1R,EGFR, PDGFRA,PDGFRB,NTRK1,NTRK3,SYK,KDR,ROS1,FGFR1,FGFR2,FGFR4,RET,KIT,TP53* |
| Negative regulation of cell death | 5.09x10^-22^ | *MPL,JAK2,AURKA,JAK3,MET,MRE11,ERBB2,ERBB3,HIF1A,IGF1R,ABL1,ANGPT1,CSF1R, BRCA1,EGFR,RB1,PDGFRB,SPP1,PIK3CA,NTRK1,ESR1,NTRK3,APC,BARD1,AKT1,KDR,SMO,MSH2,FGFR2,PTEN,KIT,EPCAM,ARAF,PALB2,NOTCH1,TP53* |
| Positive regulation of phosphorylation | 5.87x10^-22^ | *VEGFC,JAK2,MET,MRE11,STK11,ERBB2,ERBB3,HIF1A,IGF1R,ABL1,ANGPT1,CSF1R,EGFR, PDGFRA,PDGFRB,PIK3CA,NTRK1,ESR1,NTRK3,CHEK2,SYK,NBN,AKT1,KDR,FGFR1,FGFR2,ATM,FGFR4,RET,PTEN,VEGFD,KIT,ARAF,NOTCH1,TP53,RAD50* |
| Transmembrane receptor protein tyrosine kinase signaling pathway | 7.54x10^-22^ | *VEGFC,JAK2,JAK3,MET,ERBB2,ERBB3,HIF1A,IGF1R,ABL1,ANGPT1,CSF1R,EGFR,PDGFRA, PDGFRB,PIK3CA,NTRK1,ESR1,NTRK3,SYK,APC,AKT1,KDR,ROS1,FGFR1,FGFR2,FGFR4,RET,PTEN,VEGFD,KIT,NOTCH1* |
| Central carbon metabolism in cancer | 8.54x10^-22^ | *MET,ERBB2,HIF1A,EGFR,PDGFRA,PDGFRB,PIK3CA,NTRK1,NTRK3,AKT1,FGFR1,FGFR2, IDH1,RET,PTEN,KIT,TP53* |
| Transmembrane receptor protein tyrosine kinase activity | 8.57x10^-22^ | *MET,ERBB2,ERBB3,IGF1R,CSF1R,EGFR,PDGFRA,PDGFRB,NTRK1,NTRK3,KDR,ROS1,FGFR1FGFR2,FGFR4,RET,KIT* |
| Positive regulation of protein phosphorylation | 1.88x10^-21^ | *VEGFC,JAK2,MET,MRE11,STK11,ERBB2,ERBB3,IGF1R,ABL1,ANGPT1,CSF1R,EGFR,PDGFRA,PDGFRB,PIK3CA,NTRK1,ESR1,NTRK3,CHEK2,SYK,NBN,AKT1,KDR,FGFR1,FGFR2,ATM, FGFR4,RET,PTEN,VEGFD,KIT,ARAF,NOTCH1,TP53,RAD50* |
| Negative regulation of programmed cell death | 4.05x10^-21^ | *MPL,JAK2,AURKA,JAK3,MET,MRE11,ERBB2,ERBB3,HIF1A,IGF1R,ABL1,ANGPT1,CSF1R, BRCA1,EGFR,RB1,PDGFRB,SPP1,PIK3CA,NTRK1,APC,BARD1,AKT1,KDR,SMO,MSH2,FGFR2,PTEN,KIT,EPCAM,ARAF,PALB2,NOTCH1,TP53* |
| Positive regulation of phosphate metabolic process | 5.40x10^-21^ | *VEGFC,JAK2,MET,MRE11,STK11,ERBB2,ERBB3,HIF1A,IGF1R,ABL1,ANGPT1,CSF1R,EGFR, PDGFRA,PDGFRB,PIK3CA,NTRK1,ESR1,NTRK3,CHEK2,SYK,NBN,AKT1,KDR,FGFR1,FGFR2,ATM,FGFR4,RET,PTEN,VEGFD,KIT,ARAF,NOTCH1,TP53,RAD50* |
| Positive regulation of phosphorus metabolic process | 5.40x10^-21^ | *VEGFC,JAK2,MET,MRE11,STK11,ERBB2,ERBB3,HIF1A,IGF1R,ABL1,ANGPT1,CSF1R,EGFR, PDGFRA,PDGFRB,PIK3CA,NTRK1,ESR1,NTRK3,CHEK2,SYK,NBN,AKT1,KDR,FGFR1,FGFR2,ATM,FGFR4,RET,PTEN,VEGFD,KIT,ARAF,NOTCH1,TP53,RAD50* |
| Positive regulation of protein metabolic process | 1.09x10^-20^ | *VEGFC,JAK2,AURKA,MET,MRE11,STK11,ERBB2,ERBB3,IGF1R,ABL1,ANGPT1,CSF1R,FOXL2,BRCA1,EGFR,PDGFRA,PDGFRB,PIK3CA,NTRK1,ESR1,NTRK3,CHEK2,SYK,APC,BARD1,NBN,AKT1,KDR,FGFR1,FGFR2,ATM,FGFR4,RET,PTEN,VEGFD,KIT,ARAF,NOTCH1,STAT1,TP53, RAD50* |
| Enzyme linked receptor protein signaling pathway | 1.89x10^-20^ | *VEGFC,JAK1,JAK2,JAK3,MET,STK11,ERBB2,ERBB3,HIF1A,IGF1R,ABL1,ANGPT1,CSF1R, EGFR,PDGFRA,PDGFRB,PIK3CA,NTRK1,ESR1,NTRK3,SYK,APC,AKT1,KDR,ROS1,FGFR1, FGFR2,FGFR4,RET,PTEN,VEGFD,KIT,NOTCH1,TP53* |
| Transmembrane receptor protein kinase activity | 1.89x10^-20^ | *MET,ERBB2,ERBB3,IGF1R,CSF1R,EGFR,PDGFRA,PDGFRB,NTRK1,NTRK3,KDR,ROS1, FGFR1,FGFR2,FGFR4,RET,KIT* |
| ATP binding | 3.64x10^-20^ | *RAD51C,JAK1,JAK2,AURKA,JAK3,MET,STK11,MSH6,ERBB2,ERBB3,BRIP1,IGF1R,ABL1,CSF1REGFR,PDGFRA,PDGFRB,PIK3CA,NTRK1,NTRK3,CHEK2,SYK,MLH1,AKT1,KDR,ROS1,FGFR1,MSH2,MSH3,FGFR2,ATM,FGFR4,RET,KIT,ARAF,TP53,RAD50* |
| Positive regulation of protein modification process | 7.42x10^-20^ | *VEGFC,JAK2,MET,MRE11,STK11,ERBB2,ERBB3,IGF1R,ABL1,ANGPT1,CSF1R,BRCA1,EGFR, PDGFRA,PDGFRB,PIK3CA,NTRK1,ESR1,NTRK3,CHEK2,SYK,NBN,AKT1,KDR,FGFR1,FGFR2,ATM,FGFR4,RET,PTEN,VEGFD,KIT,ARAF,NOTCH1,TP53,RAD50* |
| Tube development | 8.01x10^-20^ | *VEGFC,FOXM1,JAK1,MYCN,MET,ERBB2,HIF1A,BRIP1,ABL1,NF1,ANGPT1,CSF1R,BRCA1, EGFR,PDGFRA,RB1,PDGFRB,PIK3CA,NTRK1,ESR1,SYK,AKT1,KDR,SMO,FGFR1,FGFR2, FGFR4,RET,PTEN,VEGFD,CDH1,KIT,EPCAM,NOTCH1,STAT1* |
| Positive regulation of cell population proliferation | 1.38x10^-19^ | *MPL,VEGFC,FOXM1,JAK2,MYCN,JAK3,ERBB2,ERBB3,HIF1A,IGF1R,ABL1,NF1,CSF1R,EGFR,PDGFRA,PDGFRB,PIK3CA,ESR1,NTRK3,SYK,NBN,AKT1,KDR,SMO,FGFR1,FGFR2,FGFR4, PTEN,VEGFD,KIT,EPCAM,NOTCH1,STAT1* |
| Positive regulation of developmental process | 1.38x10^-19^ | *MPL,VEGFC,JAK1,CDKN2A,JAK2,AURKA,MET,STK11,ERBB3,HIF1A,IGF1R,ABL1,NF1,CSF1R,BRCA1,PDGFRA,RB1,PDGFRB,NTRK1,NTRK3,SYK,APC,MLH1,NFKBIZ,AKT1,KDR,SMO, FGFR1,MSH2,FGFR2,FGFR4,RET,PTEN,VEGFD,KIT,NOTCH1,STAT1,TP53* |
| Adenyl ribonucleotide binding | 1.79x10^-19^ | *RAD51C,JAK1,JAK2,AURKA,JAK3,MET,STK11,MSH6,ERBB2,ERBB3,BRIP1,IGF1R,ABL1,CSF1REGFR,PDGFRA,PDGFRB,PIK3CA,NTRK1,NTRK3,CHEK2,SYK,MLH1,AKT1,KDR,ROS1,FGFR1,MSH2,MSH3,FGFR2,ATM,FGFR4,RET,KIT,ARAF,TP53,RAD50* |
| Positive regulation of cellular protein Metabolic process | 2.08x10^-19^ | *VEGFC,JAK2,AURKA,MET,MRE11,STK11,ERBB2,ERBB3,IGF1R,ABL1,ANGPT1,CSF1R,FOXL2,BRCA1,EGFR,PDGFRA,PDGFRB,PIK3CA,NTRK1,ESR1,NTRK3,CHEK2,SYK,NBN,AKT1,KDR, FGFR1,FGFR2,ATM,FGFR4,RET,PTEN,VEGFD,KIT,ARAF,NOTCH1,STAT1,TP53,RAD50* |
| Adenyl nucleotide binding | 2.29x10^-19^ | *RAD51C,JAK1,JAK2,AURKA,JAK3,MET,STK11,MSH6,ERBB2,ERBB3,BRIP1,IGF1R,ABL1,CSF1REGFR,PDGFRA,PDGFRB,PIK3CA,NTRK1,NTRK3,CHEK2,SYK,MLH1,AKT1,KDR,ROS1,FGFR1,MSH2,MSH3,FGFR2,ATM,FGFR4,RET,KIT,ARAF,TP53,RAD50* |
| Negative regulation of apoptotic process | 5.36x10^-19^ | *MPL,JAK2,AURKA,JAK3,MRE11,ERBB2,ERBB3,HIF1A,IGF1R,ABL1,ANGPT1,CSF1R,BRCA1, EGFR,RB1,PDGFRB,SPP1,PIK3CA,NTRK1,APC,BARD1,AKT1,KDR,SMO,MSH2,FGFR2,PTEN, EPCAM,ARAF,PALB2,NOTCH1,TP53* |
| PI3K-Akt signaling pathway | 5.80x10^-19^ | *VEGFC,JAK1,JAK2,JAK3,MET,STK11,IGF1R,ANGPT1,CSF1R,BRCA1,EGFR,PDGFRA,PDGFRB,SPP1,PIK3CA,SYK,AKT1,KDR,FGFR1,FGFR2,FGFR4,PTEN,VEGFD,KIT,TP53* |
| Reproductive structure development | 1.46x10^-19^ | *MET,STK11,HIF1A,BRIP1,IGF1R,ANGPT1,FOXL2,BRCA2,EGFR,PDGFRA,PDGFRB,SPP1, NTRK1,ESR1,APC,AKT1,KDR,MSH2,FGFR2,ATM,IDH1,PTEN,CDH1,KIT,NOTCH1* |
| Gland development | 1.53x10^-18^ | *FOXM1,JAK2,AURKA,MET,STK11,HIF1A,IGF1R,ABL1,NF1,CSF1R,BRCA2,EGFR,PDGFRA, PDGFRB,PIK3CA,ESR1,APC,AKT1,SMO,FGFR1,FGFR2,ATM,PTEN,CDH1,NOTCH1* |
| Regulation of transferase activity | 1.72x10^-18^ | *VEGFC,CDKN2A,JAK2,MET,MRE11,STK11,ERBB2,ERBB3,IGF1R,ABL1,NF1,ANGPT1,CSF1R, EGFR,PDGFRA,RB1,PDGFRB,PIK3CA,NTRK1,NTRK3,SYK,APC,NBN,AKT1,FGFR1,ATM,RET, PTEN,KIT,ARAF,TP53,RAD50* |
| Reproductive system development | 1.77x10^-18^ | *MET,STK11,HIF1A,BRIP1,IGF1R,ANGPT1,FOXL2,BRCA2,EGFR,PDGFRA,PDGFRB,SPP1, NTRK1,ESR1,APC,AKT1,KDR,MSH2,FGFR2,ATM,IDH1,PTEN,CDH1,KIT,NOTCH1* |
| Drug binding | 1.81x10^-18^ | *RAD51C,JAK1,JAK2,AURKA,JAK3,MET,STK11,MSH6,ERBB2,ERBB3,BRIP1,IGF1R,ABL1,CSF1REGFR,PDGFRA,PDGFRB,PIK3CA,NTRK1,NTRK3,CHEK2,SYK,MLH1,AKT1,KDR,SMO,ROS1,FGFR1,MSH2,MSH3,FGFR2,ATM,FGFR4,RET,KIT,ARAF,TP53,RAD50* |
| EGFR tyrosine kinase inhibitor resistance | 2.17x10^-18^ | *JAK1,JAK2,MET,ERBB2,ERBB3,IGF1R,NF1,EGFR,PDGFRA,PDGFRB,PIK3CA,AKT1,KDR, FGFR2,PTEN,ARAF* |
| Purine ribonucleoside triphosphate binding | 5.35x10^-18^ | *RAD51C,JAK1,JAK2,AURKA,JAK3,MET,STK11,MSH6,TUBB3,ERBB2,ERBB3,BRIP1,IGF1R, ABL1,CSF1R,EGFR,PDGFRA,PDGFRB,PIK3CA,NTRK1,NTRK3,CHEK2,SYK,MLH1,AKT1,KDR,ROS1,FGFR1,MSH2,MSH3,FGFR2,ATM,FGFR4,RET,KIT,ARAF,TP53,RAD50* |
| Peptidyl-amino acid modification | 7.32x10^-18^ | *VEGFC,JAK1,JAK2,AURKA,JAK3,MET,STK11,ERBB2,ERBB3,IGF1R,ABL1,ANGPT1,CSF1R, BRCA1,BRCA2,EGFR,PDGFRA,PDGFRB,PIK3CA,NTRK1,NTRK3,CHEK2,SYK,AKT1,KDR,ROS1,FGFR1,FGFR2,ATM,FGFR4,RET,KIT,ARAF,TP53* |
| MAPK cascade | 8.07x10^-18^ | *FOXM1,JAK1,JAK2,JAK3,MET,ERBB2,ERBB3,IGF1R,ABL1,NF1,ANGPT1,CSF1R,EGFR, PDGFRA,PDGFRB,NTRK1,ESR1,NTRK3,SYK,APC,AKT1,KDR,ROS1,FGFR1,FGFR2,FGFR4, RET,PTEN,KIT,ARAF,NOTCH1* |
| Signal transduction by protein phosphorylation | 1.13x10^-17^ | *FOXM1,JAK1,JAK2,JAK3,MET,ERBB2,ERBB3,IGF1R,ABL1,NF1,ANGPT1,CSF1R,EGFR, PDGFRA,PDGFRB,NTRK1,ESR1,NTRK3,SYK,APC,AKT1,KDR,ROS1,FGFR1,FGFR2,FGFR4,RE,PTEN,KIT,ARAF,NOTCH1* |
| Positive regulation of catalytic activity | 1.40x10^-17^ | *VEGFC,JAK2,MET,MRE11,STK11,MSH6,ERBB2,ERBB3,HIF1A,IGF1R,ABL1,NF1,ANGPT1,CSF1R,FOXL2,EGFR,PDGFRA,PDGFRB,PIK3CA,NTRK1,ESR1,NTRK3,SYK,NBN,AKT1,FGFR1,MSH2MSH3,FGFR2,FGFR4,RET,PTEN,KIT,ARAF,STAT1,RAD50* |
| Regulation of kinase activity | 1.62x10^-17^ | *VEGFC,CDKN2A,JAK2,MET,MRE11,STK11,ERBB2,ERBB3,IGF1R,ABL1,NF1,ANGPT1,CSF1R, EGFR,PDGFRA,RB1,PDGFRB,PIK3CA,NTRK1,NTRK3,SYK,APC,NBN,AKT1,FGFR1,RET,PTEN,KIT,ARAF,RAD50* |
| Phosphatidylinositol-mediated signaling | 2.50x10^-17^ | *JAK2,ERBB2,ERBB3,IGF1R,NF1,ANGPT1,CSF1R,EGFR,PDGFRA,PDGFRB,PIK3CA,NTRK1, NTRK3,AKT1,KDR,FGFR1,PTEN,KIT* |
| Regulation of protein kinase activity | 2.61x10^-17^ | *VEGFC,CDKN2A,JAK2,MET,MRE11,STK11,ERBB2,ERBB3,IGF1R,ABL1,NF1,ANGPT1,CSF1R, EGFR,RB1,PDGFRB,PIK3CA,NTRK1,NTRK3,SYK,APC,NBN,AKT1,FGFR1,RET,PTEN,KIT,ARAF,RAD50* |
| Purine ribonucleotide binding | 2.78x10^-17^ | *RAD51C,JAK1,JAK2,AURKA,JAK3,MET,STK11,MSH6,TUBB3,ERBB2,ERBB3,BRIP1,IGF1R, ABL1,CSF1R,EGFR,PDGFRA,PDGFRB,PIK3CA,NTRK1,NTRK3,CHEK2,SYK,MLH1,AKT1,KDR,ROS1,FGFR1,MSH2,MSH3,FGFR2,ATM,FGFR4,RET,KIT,ARAF,TP53,RAD50* |
| Protein kinase B signaling | 3.05x10^-17^ | *MET,STK11,ERBB2,ERBB3,IGF1R,ANGPT1,EGFR,PDGFRA,PDGFRB,PIK3CA,ESR1,MC1R, AKT1,KDR,FGFR1,FGFR2,FGFR4,RET,PTEN,KIT* |
| Inositol lipid-mediated signaling | 3.30x10^-17^ | *JAK2,ERBB2,ERBB3,IGF1R,NF1,ANGPT1,CSF1R,EGFR,PDGFRA,PDGFRB,PIK3CA,NTRK1, NTRK3,AKT1,KDR,FGFR1,PTEN,KIT* |
| Phosphatidylinositol 3-kinase signaling | 3.39x10^-17^ | *JAK2,ERBB2,ERBB3,IGF1R,NF1,ANGPT1,EGFR,PDGFRA,PDGFRB,PIK3CA,NTRK1,NTRK3, AKT1,KDR,FGFR1,PTEN,KIT* |
| Ribonucleotide binding | 3.77x10^-17^ | *RAD51C,JAK1,JAK2,AURKA,JAK3,MET,STK11,MSH6,TUBB3,ERBB2,ERBB3,BRIP1,IGF1R, ABL1,CSF1R,EGFR,PDGFRA,PDGFRB,PIK3CA,NTRK1,NTRK3,CHEK2,SYK,MLH1,AKT1,KDR,ROS1,FGFR1,MSH2,MSH3,FGFR2,ATM,FGFR4,RET,KIT,ARAF,TP53,RAD50* |
| Purine nucleotide binding | 4.05x10^-17^ | *RAD51C,JAK1,JAK2,AURKA,JAK3,MET,STK11,MSH6,TUBB3,ERBB2,ERBB3,BRIP1,IGF1R, ABL1,CSF1R,EGFR,PDGFRA,PDGFRB,PIK3CA,NTRK1,NTRK3,CHEK2,SYK,MLH1,AKT1,KDR,ROS1,FGFR1,MSH2,MSH3,FGFR2,ATM,FGFR4,RET,KIT,ARAF,TP53,RAD50* |
| Circulatory system development | 4.80x10^-17^ | *VEGFC,FOXM1,JAK1,MET,STK11,ERBB2,ERBB3,HIF1A,ABL1,NF1,ANGPT1,BRCA1,EGFR, PDGFRA,RB1,PDGFRB,PIK3CA,NTRK1,NTRK3,SYK,APC,AKT1,KDR,SMO,FGFR1,FGFR2,ATMPTEN,VEGFD,KIT,NOTCH1,STAT1,TP53* |
| Immune system development | 6.75x10^-17^ | *MPL,JAK2,JAK3,STK11,MSH6,ERBB2,HIF1A,ABL1,NF1,ANGPT1,CSF1R,BRCA2,PDGFRA,RB1,PDGFRB,NTRK1,SYK,APC,MLH1,NBN,NFKBIZ,KDR,MSH2,MSH3,FGFR2,ATM,RET,KIT, NOTCH1,STAT1,TP53* |
| Regulation of MAPK cascade | 9.69x10^-17^ | *FOXM1,JAK2,MET,ERBB2,IGF1R,ABL1,NF1,ANGPT1,CSF1R,EGFR,PDGFRA,PDGFRB,NTRK1,ESR1,NTRK3,SYK,APC,AKT1,KDR,ROS1,FGFR1,FGFR2,FGFR4,RET,PTEN,KIT,ARAF,NOTCH1* |
| Tube morphogenesis | 1.06x10^-16^ | *VEGFC,FOXM1,JAK1,MYCN,MET,ERBB2,HIF1A,ABL1,NF1,ANGPT1,CSF1R,BRCA1,EGFR, PDGFRA,PDGFRB,PIK3CA,NTRK1,ESR1,SYK,AKT1,KDR,SMO,FGFR1,FGFR2,RET,PTEN, VEGFD,CDH1,NOTCH1,STAT1* |
| Regulation of protein kinase B signaling | 1.23x10^-16^ | *MET,STK11,ERBB2,ERBB3,IGF1R,ANGPT1,EGFR,PDGFRA,PDGFRB,PIK3CA,ESR1,MC1R, AKT1,FGFR1,FGFR2,FGFR4,RET,PTEN,KIT* |
| Epithelium development | 1.37x10^-16^ | *VEGFC,JAK2,MYCN,MET,HIF1A,IGF1R,ABL1,NF1,ANGPT1,CSF1R,FOXL2,BRCA2,EGFR, PDGFRA,NTRK1,ESR1,NTRK3,APC,AKT1,KDR,SMO,ROS1,FGFR1,FGFR2,ATM,FGFR4,RET, PTEN,CDH1,EPCAM,PALB2,NOTCH1,STAT1,TP53* |
| Developmental process involved in reproduction | 2.46x10^-16^ | *AURKA,MET,STK11,HIF1A,BRIP1,IGF1R,ANGPT1,FOXL2,BRCA2,EGFR,PDGFRA,PDGFRB, SPP1,NTRK1,ESR1,APC,MLH1,AKT1,KDR,MSH2,FGFR2,ATM,IDH1,PTEN,CDH1,KIT,NOTCH1* |
| Cell cycle process | 2.52x10^-16^ | *FOXM1,RAD51C,CDKN2A,AURKA,MET,MRE11,STK11,MSH6,TUBB3,BRIP1,IGF1R,ABL1, BRCA1,BRCA2,EGFR,RB1,PDGFRB,ESR1,CHEK2,APC,BARD1,MLH1,NBN,AKT1,FGFR1, MSH2,MSH3,FGFR2,ATM,XPO1,PTEN,RRM2,NOTCH1,TP53,RAD50* |
| DNA metabolic process | 2.68x10^-16^ | *FOXM1,RAD51C,MET,MRE11,MSH6,BRIP1,IGF1R,ABL1,FOXL2,BRCA1,BRCA2,EGFR, PDGFRB,PIK3CA,MC1R,CHEK2,BARD1,MLH1,NBN,AKT1,FGFR1,MSH2,MSH3,ATM,FGFR4, RRM2,PALB2,MUTYH,TP53,RAD50* |
| Regulation of cell cycle | 3.14x10^-16^ | *FOXM1,RAD51C,CDKN2A,AURKA,MET,MRE11,STK11,MSH6,BRIP1,IGF1R,ABL1,BRCA1, BRCA2,EGFR,RB1,PDGFRB,ESR1,CHEK2,APC,BARD1,NBN,AKT1,FGFR1,MSH2,FGFR2,ATM,XPO1,PTEN,RRM2,NOTCH1,TP53,RAD50* |
| Organelle fission | 3.61x10^-16^ | *FOXM1,RAD51C,CDKN2A,AURKA,MET,MRE11,MSH6,TUBB3,BRIP1,IGF1R,ABL1,BRCA1, BRCA2,EGFR,RB1,PDGFRB,ESR1,CHEK2,APC,MLH1,NBN,AKT1,KDR,FGFR1,MSH2,MSH3, FGFR2,ATM,PTEN,RRM2,TP53,RAD50* |
| Reproductive process | 3.65x10^-16^ | *RAD51C,AURKA,MET,MRE11,STK11,MSH6,ERBB2,HIF1A,BRIP1,IGF1R,ANGPT1,FOXL2, BRCA2,EGFR,PDGFRA,PDGFRB,SPP1,NTRK1,ESR1,APC,MLH1,NBN,AKT1,KDR,ROS1,MSH2,MSH3,FGFR2,ATM,IDH1,PTEN,CDH1,KIT,NOTCH1,RAD50* |
| Reproduction | 3.88x10^-16^ | *RAD51C,AURKA,MET,MRE11,STK11,MSH6,ERBB2,HIF1A,BRIP1,IGF1R,ANGPT1,FOXL2, BRCA2,EGFR,PDGFRA,PDGFRB,SPP1,NTRK1,ESR1,APC,MLH1,NBN,AKT1,KDR,ROS1,MSH2,MSH3,FGFR2,ATM,IDH1,PTEN,CDH1,KIT,NOTCH1,RAD50* |
| Positive regulation of kinase activity | 3.89x10^-16^ | *VEGFC,JAK2,MET,MRE11,STK11,ERBB2,ERBB3,IGF1R,ABL1,ANGPT1,CSF1R,EGFR,PDGFRA,PDGFRB,PIK3CA,NTRK1,NTRK3,SYK,NBN,AKT1,FGFR1,RET,KIT,ARAF,RAD50* |
| Positive regulation of transferase activity | 3.98x10^-16^ | *VEGFC,JAK2,MET,MRE11,STK11,ERBB2,ERBB3,IGF1R,ABL1,ANGPT1,CSF1R,EGFR,PDGFRA,PDGFRB,PIK3CA,NTRK1,NTRK3,SYK,NBN,AKT1,FGFR1,RET,PTEN,KIT,ARAF,RAD50* |
| Melanoma | 8.31x10^-16^ | *CDKN2A,MET,IGF1R,EGFR,PDGFRA,RB1,PDGFRB,PIK3CA,AKT1,FGFR1,PTEN,CDH1,ARAF,TP53* |
| Positive regulation of protein kinase activity | 1.50x10^-15^ | *VEGFC,JAK2,MET,MRE11,STK11,ERBB2,ERBB3,IGF1R,ABL1,ANGPT1,CSF1R,EGFR,PDGFRB,PIK3CA,NTRK1,NTRK3,SYK,NBN,AKT1,FGFR1,RET,KIT,ARAF,RAD50* |
| Nuclear division | 1.56x10^-15^ | *FOXM1,RAD51C,CDKN2A,AURKA,MET,MRE11,MSH6,TUBB3,BRIP1,IGF1R,ABL1,BRCA1, BRCA2,EGFR,RB1,PDGFRB,ESR1,CHEK2,APC,MLH1,NBN,AKT1,FGFR1,MSH2,MSH3,FGFR2,ATM,PTEN,RRM2,TP53,RAD50* |
| Signaling by PDGF | 2.43x10^-15^ | *JAK1,JAK2,JAK3,MET,ERBB2,ERBB3,NF1,ANGPT1,EGFR,PDGFRA,PDGFRB,SPP1,PIK3CA, AKT1,FGFR1,FGFR2,FGFR4,RET,PTEN,KIT,ARAF,STAT1,TP53* |
| Regulation of phosphatidylinositol 3-kinase signaling | 3.01x10^-15^ | *JAK2,ERBB2,ERBB3,IGF1R,ANGPT1,EGFR,PDGFRA,PDGFRB,PIK3CA,NTRK1,NTRK3,KDR, FGFR1,PTEN,KIT* |
| Signaling by SCF-KIT | 3.96x10^-15^ | *JAK1,JAK2,JAK3,MET,ERBB2,ERBB3,NF1,ANGPT1,EGFR,PDGFRA,PDGFRB,PIK3CA,AKT1, FGFR1,FGFR2,FGFR4,RET,PTEN,KIT,ARAF,STAT1,TP53* |
| Signaling by VEGF | 4.22x10^-15^ | *VEGFC,JAK1,JAK2,JAK3,MET,ERBB2,ERBB3,NF1,ANGPT1,EGFR,PDGFRA,PDGFRB,PIK3CA,AKT1,KDR,FGFR1,FGFR2,FGFR4,RET,VEGFD,KIT,ARAF* |
| Positive regulation of cell motility | 5.03x10^-15^ | *VEGFC,JAK2,MET,HIF1A,IGF1R,ABL1,ANGPT1,CSF1R,EGFR,PDGFRA,PDGFRB,NTRK3,APC,AKT1,KDR,SMO,FGFR1,ATM,RET,VEGFD,KIT,EPCAM,NOTCH1,TP53* |
| Regulation of epithelial cell proliferation | 5.47x10^-15^ | *VEGFC,STK11,ERBB2,HIF1A,NF1,ANGPT1,BRCA2,EGFR,RB1,ESR1,APC,AKT1,KDR,SMO, FGFR1,FGFR2,PTEN,VEGFD,CDH1,NOTCH1,STAT1* |
| Epithelial cell proliferation | 5.65x10^-15^ | *VEGFC,STK11,ERBB2,HIF1A,NF1,ANGPT1,BRCA2,EGFR,RB1,ESR1,APC,AKT1,KDR,SMO, FGFR1,FGFR2,PTEN,VEGFD,CDH1,KIT,NOTCH1,STAT1* |
| IRS-related events triggered by IGF1R | 6.09x10^-15^ | *JAK1,JAK2,JAK3,MET,STK11,ERBB2,ERBB3,IGF1R,NF1,ANGPT1,EGFR,PDGFRA,PDGFRB, PIK3CA,AKT1,FGFR1,FGFR2,FGFR4,RET,KIT,ARAF* |
| IGF1R signaling cascade | 6.09x10^-15^ | *JAK1,JAK2,JAK3,MET,STK11,ERBB2,ERBB3,IGF1R,NF1,ANGPT1,EGFR,PDGFRA,PDGFRB, PIK3CA,AKT1,FGFR1,FGFR2,FGFR4,RET,KIT,ARAF* |
| Signaling by Type 1 IGF1R | 6.52x10^-15^ | *JAK1,JAK2,JAK3,MET,STK11,ERBB2,ERBB3,IGF1R,NF1,ANGPT1,EGFR,PDGFRA,PDGFRB, PIK3CA,AKT1,FGFR1,FGFR2,FGFR4,RET,KIT,ARAF* |
| Positive regulation of signal transduction | 8.77x10^-15^ | *JAK1,JAK2,JAK3,MET,STK11,ERBB2,ERBB3,HIF1A,IGF1R,ABL1,NF1,ANGPT1,CSF1R,EGFR, PDGFRA,PDGFRB,PIK3CA,NTRK1,ESR1,NTRK3,MC1R,SYK,AKT1,KDR,SMO,FGFR1,FGFR2, ATM,FGFR4,RET,PTEN,KIT,ARAF,NOTCH1,TP53* |
| Positive regulation of intracellular signal transduction | 9.09x10^-15^ | *JAK2,MET,ERBB2,ERBB3,IGF1R,ABL1,ANGPT1,CSF1R,EGFR,PDGFRA,PDGFRB,PIK3CA, NTRK1,ESR1,NTRK3,MC1R,SYK,AKT1,KDR,FGFR1,FGFR2,ATM,FGFR4,RET,PTEN,KIT,ARAF, NOTCH1,TP53* |
| Regulation of cell motility | 9.31x10^-15^ | *VEGFC,JAK2,MET,ERBB2,ERBB3,HIF1A,IGF1R,ABL1,NF1,ANGPT1,CSF1R,EGFR,PDGFRA, PDGFRB,NTRK3,APC,AKT1,KDR,SMO,FGFR1,ATM,RET,PTEN,VEGFD,CDH1,KIT,EPCAM, NOTCH1,TP53* |
| Downstream signal transduction | 9.99x10^-15^ | *JAK1,JAK2,JAK3,MET,ERBB2,ERBB3,NF1,ANGPT1,EGFR,PDGFRA,PDGFRB,PIK3CA,AKT1, FGFR1,FGFR2,FGFR4,RET,PTEN,KIT,ARAF,STAT1,TP53* |
| Positive regulation of cellular component movement | 1.16x10^-14^ | *VEGFC,JAK2,MET,HIF1A,IGF1R,ABL1,ANGPT1,CSF1R,EGFR,PDGFRA,PDGFRB,NTRK3,APC,AKT1,KDR,SMO,FGFR1,ATM,RET,VEGFD,KIT,EPCAM,NOTCH1,TP53* |
| DAP12 signaling | 1.27x10^-14^ | *JAK1,JAK2,JAK3,MET,ERBB2,ERBB3,NF1,ANGPT1,EGFR,PDGFRA,PDGFRB,PIK3CA,SYK, AKT1,FGFR1,FGFR2,FGFR4,RET,PTEN,KIT,ARAF,TP53* |
| Positive regulation of protein kinase B signaling | 1.36x10^-14^ | *MET,ERBB2,ERBB3,IGF1R,ANGPT1,EGFR,PDGFRA,PDGFRB,PIK3CA,ESR1,MC1R,FGFR1, FGFR2,FGFR4,RET,KIT* |
| Positive regulation of locomotion | 1.58x10^-14^ | *VEGFC,JAK2,MET,HIF1A,IGF1R,ABL1,ANGPT1,CSF1R,EGFR,PDGFRA,PDGFRB,NTRK3,APC,AKT1,KDR,SMO,FGFR1,ATM,RET,VEGFD,KIT,EPCAM,NOTCH1,TP53* |
| Positive regulation of MAPK cascade | 2.16x10^-14^ | *JAK2,MET,ERBB2,IGF1R,ABL1,ANGPT1,CSF1R,EGFR,PDGFRA,PDGFRB,NTRK1,ESR1, NTRK3,SYK,KDR,FGFR1,FGFR2,FGFR4,RET,PTEN,KIT,ARAF,NOTCH1* |
| DAP12 interactions | 3.03x10^-14^ | *JAK1,JAK2,JAK3,MET,ERBB2,ERBB3,NF1,ANGPT1,EGFR,PDGFRA,PDGFRB,PIK3CA,SYK, AKT1,FGFR1,FGFR2,FGFR4,RET,PTEN,KIT,ARAF,TP53* |
| Positive regulation of cell migration | 3.09x10^-14^ | *VEGFC,JAK2,MET,HIF1A,IGF1R,ABL1,ANGPT1,CSF1R,EGFR,PDGFRA,PDGFRB,NTRK3,APC,AKT1,KDR,SMO,FGFR1,ATM,RET,VEGFD,KIT,NOTCH1,TP53* |
| Protein serine/threonine kinase activity | 3.18x10^-14^ | *CDKN2A,JAK2,AURKA,MET,STK11,ERBB2,IGF1R,ABL1,NF1,CSF1R,EGFR,RB1,PDGFRB, PIK3CA,NTRK1,NTRK3,CHEK2,SYK,APC,AKT1,FGFR1,ATM,RET,PTEN,KIT,ARAF* |
| Positive regulation of phosphatidylinositol 3-kinase signaling | 3.86x10^-14^ | *JAK2,ERBB2,ERBB3,IGF1R,ANGPT1,PDGFRA,PDGFRB,PIK3CA,NTRK1,NTRK3,KDR,FGFR1, KIT* |

Pathway enrichment analysis was performed using ToppFun in the ToppGene Suite to analyze functionally the genes of interest identified in the 31 breast cancers.

^*^Pathways with a Bonferroni *q* value ≤ 0.01 were selected as top pathways
